# Adhesion of Active Cytoskeletal Vesicles

**DOI:** 10.1016/j.bpj.2018.10.013

**Published:** 2018-10-23

**Authors:** Renu Maan, Etienne Loiseau, Andreas R. Bausch

**Affiliations:** 1 Lehrstuhl für Biophysik E27, Physik-Department, Technische Universität München, Garching, Germany; 2 Department of Bionanoscience, Kavli Institute of NanoScience, Faculty of Applied Sciences, Delft University of Technology, Delft, the Netherlands; 3 Aix-Marseille Université, CNRS, CINAM, Marseille, France

## Abstract

Regulation of adhesion is a ubiquitous feature of living cells, observed during processes such as motility, antigen recognition, or rigidity sensing. At the molecular scale, a myriad of mechanisms are necessary to recruit and activate the essential proteins, whereas at the cellular scale, efficient regulation of adhesion relies on the cell’s ability to adapt its global shape. To understand the role of shape remodeling during adhesion, we use a synthetic biology approach to design a minimal experimental model, starting with a limited number of building blocks. We assemble cytoskeletal vesicles whose size, reduced volume, and cytoskeletal contractility can be independently tuned. We show that these cytoskeletal vesicles can sustain strong adhesion to solid substrates only if the actin cortex is actively remodeled significantly. When the cytoskeletal vesicles are deformed under hypertonic osmotic pressure, they develop a crumpled geometry with deformations. In the presence of molecular motors, these deformations are dynamic in nature, and the excess membrane area generated thereby can be used to gain adhesion energy. The cytoskeletal vesicles are able to attach to the rigid glass surfaces even under strong adhesive forces just like the cortex-free vesicles. The balance of deformability and adhesion strength is identified to be key to enable cytoskeletal vesicles to adhere to solid substrates.

## Introduction

Giant unilamellar vesicles have proven to be an excellent model system to study basic processes of cellular adhesion (1, 2, 3, 4, 5, 6, 7). The interactions involved in the formation of adhesion domains and the fundamental differences between cell-cell and cell-substrate adhesion have been identified (8). Recently, it has been shown that adhering vesicles act as force generators and that the adhesion process itself is sufficient to induce traction forces on a surface (9). The adhesion forces can be well controlled by the membrane composition of the vesicle and surface functionalization (10). During adhesion strengthening, the adhesion forces pull on the membrane, dampen its fluctuations, and thus increase the membrane tension (11). An increase in adhesion strength above a critical value causes the membrane tension to reach its critical lysis tension, leading to vesicle bursting (12). Under conditions of specific adhesion, the lateral forces from the surface come from the attraction between the membrane-bound receptors and ligands on the surface. In going from an unbound state to a bound state, the vesicles undergo significant shape transformations (11, 12, 13, 14). This adhesion-induced shape transformation has been successfully explained by free energy minimization in the framework of the Helfrich theory of elastic cells (15, 16). Thus far, insight into the adhesion process through the model system lacks involvement of membrane-cytoskeletal coupling. What has already been established is that binding the cortex to the membrane causes dampening of membrane fluctuations and increases the membrane tension for both cells and vesicles (17, 18). But how this would influence the adhesion process is yet to be explored.

Here, we elucidate the role of the presence of a cytoskeletal cortex on the adhesion process in biomimetic systems. The specific adhesion of vesicles to a glass surface is mediated by biotin and streptavidin as the ligand-receptor pair. We observe that for a given ligand-receptor density for which cortex-free vesicles show strong adhesion, cytoskeletal vesicles burst. We show that the observation of bursting cytoskeletal vesicles compared to stably adhered cortex-free vesicles is due to the need for a vesicle to deform to accommodate with the surface so that it can gain in adhesion, which in turn requires excess membrane area. However, a significant amount of the excess membrane area in cytoskeletal vesicles is pinned to a rigid cortex and hence not available for the deformations to gain adhesion energy. The adhesion process relies on the availability of excess membrane area.

Because the coupling of cytoskeleton to the membrane is opposing the vesicle deformation to gain adhesion, we provide the cytoskeletal vesicles with excess membrane area by applying additional hypertonic stress. Under the reduced volume condition, the vesicles can then develop large deformations. Our experiments show that only active remodeling of the cortex can provide the required excess membrane area to deform and gain adhesion energy. Using myosin motors, we induce active remodeling in actin cortex. Under hypertonic stress, the weakly adhered active cytoskeletal vesicles make a transition from weak adhesion to strong adhesion regime without rupturing their membrane. Hence, we show that active remodeling of the cortex is thus an important component to enable the adhesion of cytoskeletal vesicles.

## Materials and Methods

### Reagents

Egg L-*α*-phosphatidylcholine lipids were ordered from Sigma-Aldrich (St. Louis, MO) (P3556) in powder form and dissolved at 50 mg/mL in a chloroform/methanol mixture (9:1, v/v). 1,2-dioleoyl-sn-glyero-3-[(N-(5-amino-1-carboxypentyl)iminodiacetic acid)succinyl) (nickel salt) lipids (Ni-NTA) (790404 C); 1,2-distearoyl-sn-glycero-3-phosphoethanolamine-N-biotinyl(polyethylene glycol)-2000] (ammonium salt) (880129 C) and PEG2000PE (880160 C) lipids were ordered from Avanti Polar Lipids (Alabaster, AL). The mineral oil was from Sigma-Aldrich (M3516), and the silicone oil (viscosity 50 centistokes) was from Roth (4020.1). Decane was from Sigma-Aldrich (D901). Biotinylated bovine serum albumin (BSA) (A8549) and streptavidin (S4762) were also purchased from Sigma-Aldrich.

### Proteins

Proteins were purified according to previously published protocols. G-actin (19, 20) and muscle myosin II (21) were purified from rabbit skeletal muscle. The fragment of *Xenopus laevis* anillin spanning amino acids 1–428 (22), excluding the myosin binding site, was cloned into a pET-28a vector and purified from *Escherichia coli* with histidine (His) tags on both termini. Anillin is a monomer with two F-actin binding sites that enables it to bundle the actin filaments (23, 24). Anillin 1–428 was stored at −80°C in buffer with 25 mM imidazole (pH 6), 25 mM KCl, 4 mM MgCl2, 1 mM EGTA, and 1 mM 1,4-dithiothreitol. The His tag on the two termini of anillin couples the actin to the Ni-NTA (nitrilotriacetic acid) lipids in the membrane.

### Buffer solution

We mixed the solution to be encapsulated on ice immediately before vesicle production. The reaction mix that was encapsulated inside the vesicle contained 1.5 *μ*M anillin, 0.1 *μ*M myosin II, 10 *μ*M G-actin, 10 mM imidazole, 1 mM MgCl2, 1 mM ATP, 1 mM EGTA, 30 mM KCl, 2 mM dithiothreitol, 300 mM sucrose, and 0.5 *μ*M Alexa Fluor 488 phalloidin. The pH of the final inside solution was 7.2. The outside solution for production of vesicles consisted only of glucose dissolved in Millipore water (Merck Millipore, Burlington, MA). The osmotic pressure of the outside solution was adjusted to be 10–15 milliosmoles (mOsm) higher than the protein mix to form stable vesicles.

### Vesicle production

Vesicles were produced using continuous droplet interface crossing encapsulation (cDICE) (25). Briefly, this method consists of a cylindrical rotating chamber successively filled with a glucose solution to collect the vesicles, a lipid-in-oil solution to saturate the oil-water interfaces, and decane as the continuous phase in which droplets were produced. The protocol to disperse the lipids in the oil solution published elsewhere (26) was modified to encapsulate proteins inside the vesicles (18). The lipid-in-oil mix contained 14% (v/v) mineral oil, 80% (v/v) silicon oil, and 6% decane. Lipids from stocks solutions (in chloroform) were first dissolved in decane and then oil (silicon + mineral) were added to give the final lipid concentration of 0.5 mM.

The reaction mix containing the cytoskeletal elements was injected through a glass capillary tube by inserting the capillary’s tip (diameter of 20 *μ*m) in decane. Because of the shear force droplets detach from the tip and are then carried by the centrifugal force through the lipid-in-oil solution, where they were first coated by a lipid monolayer and then by a second lipid monolayer while crossing the oil-water interface. The two monolayers zipped together to form a bilayer. Vesicles were collected in the glucose solution, which was sucked with a micropipette once the chamber was stopped. For the process to succeed, the osmolarity of the encapsulated solution has to be 10–15 mOsm lower than the outside. The whole process was completed in a cold room maintained at 5°C to prevent fast polymerization of the cytoskeleton. We produced vesicles in a span of 2 min. Although cDICE is a high-yield method resulting in hundreds of vesicles under most conditions, encapsulating proteins at high concentrations (10 *μ*M actin and up to 1.5 *μ*M anillin) resulted in a decrease of the yield. At the highest protein concentrations, a 100-*μ*L sample contained ∼50 vesicles with diameters ranging from 15 to 30 *μ*m. Large vesicles with a diameter of 40 *μ*m were produced using capillaries with tip diameters larger than 20 *μ*m. The lipid bilayer of the vesicles consisted of egg L-*α*-phosphatidylcholine with 10 mol % Ni-NTA and 1 mol % biotinylated PEG2000 lipids.

### Adhesion protocol

BSA-biotin and streptavidin were used to functionalize the coverslips to specifically adhere the vesicles. The stocks and working solutions of BSA, BSA-biotin, and streptavidin were all prepared in 1× phosphate-buffered saline (PBS) containing 2.7 mM KCl and 137 mM NaCl with pH 7.4 at room temperature (P4417; Sigma-Aldrich). To functionalize the coverslips, they were first incubated for 20 min at room temperature with a mix of 1 mg/mL BSA-biotin and 1 mg/mL BSA in different ratios, followed by a couple washes with 1× PBS and then further incubation with 0.5 mg/mL streptavidin. Three different v/v ratios (70:30, 50:50, and 35:65) of 1 mg/mL BSA-biotin and 1 mg/mL BSA were used in our experiments to vary the ligand density at the surface. PEG-biotin lipids in the membrane were kept at 1 mol % for the strong adhesion. After streptavidin binding, the coverslips were rinsed with the external buffer of the vesicle suspension to avoid any osmotic pressure changes caused by the PBS. Because the external buffer is just glucose in water, 5 *μ*M KCl was added to the buffer to screen short-range repulsive electrostatic interactions and allow biotin-streptavidin binding.

In addition to lowering the percentage ratio of BSA-biotin and BSA, we also added 0.5 mol % PEG2000 lipids into the vesicle membrane to lower the adhesion strength between the coverslip and the membrane.

### Deflating protocol

Vesicles were deflated by adjusting the surrounding osmotic pressure in a diffusion chamber. The chamber consisted of two compartments made of flat o-rings and separated by a membrane (Merck Millipore) with a pore size of 0.22 *μ*m. The o-rings used were of 20 mm in diameter and 2 mm in thickness.

The vesicles were confined in the bottom compartment, and their surrounding osmotic pressure was changed by adding glucose buffer in the top compartment. The osmotic pressure equilibrates in both chambers via glucose diffusion through the polycarbonate membrane separating the two compartments. The increase in osmotic pressure in the bottom compartment was followed by harvesting aliquots from the top compartment every 10 min. After the osmotic pressure measurement, which took around 30 s, the aliquots were put back in the top chamber to avoid volume differences. The calibration chart can be seen in Fig. S1.

### Imaging and analysis

Vesicles were imaged with a Leica Microscope DMI3000 B and a 63× numerical aperture 1.3 oil immersion objective for bright-field microscopy and epifluorescence, in combination with a Hamamatsu ORCA-ER camera (Hamamatsu, Japan).

Confocal images were acquired using Leica TSC SP5 and a 63× numerical aperture 1.4 oil immersion objective. The three-dimensional (3D) reconstruction using the confocal stack was done using Imaris Software. Kymographs were prepared from the time-lapse images of the vesicles sedimented on passivated surface in epifluorescence using a Fiji (27) plugin. We always used the closed chambers to image the vesicles to avoid large scale drifts and convection.

## Results and Discussion

### Cytoskeletal vesicles

**Figure 1:**
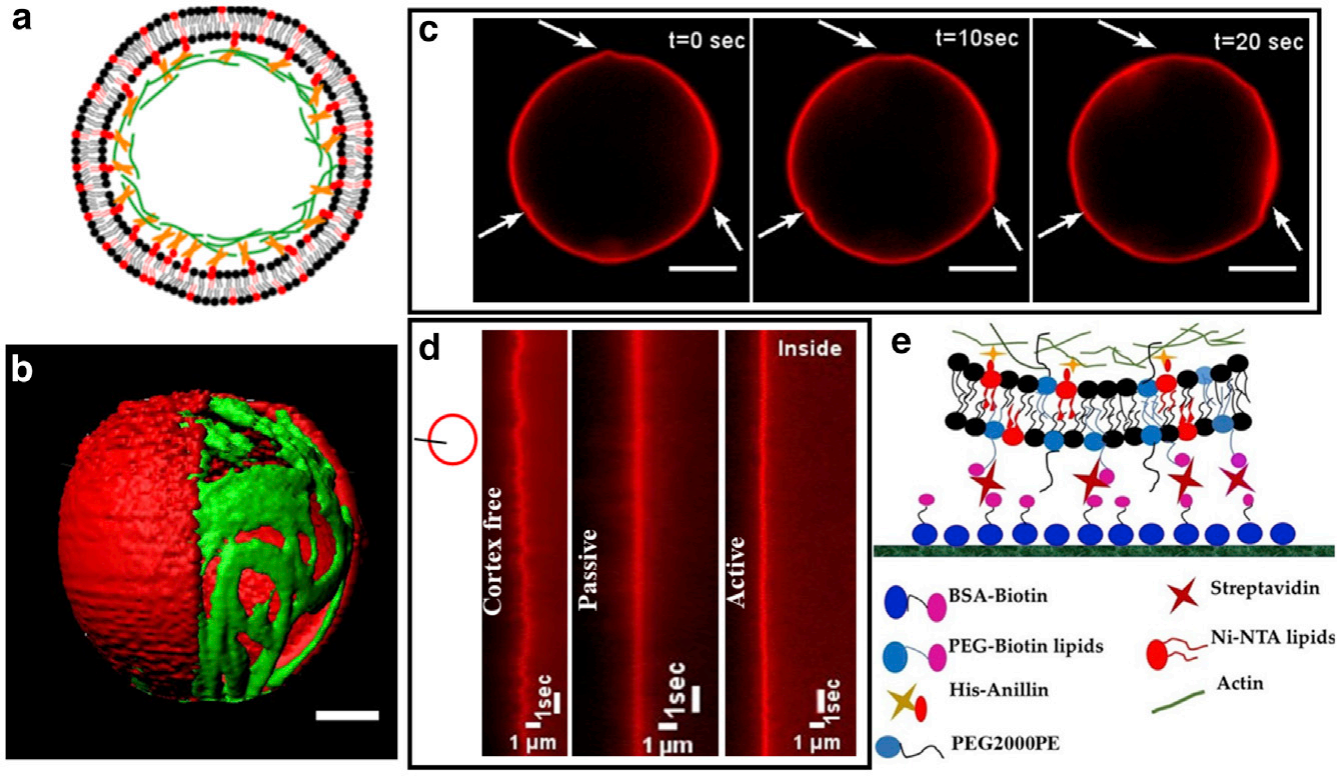
(*a*) The lipid membrane contains a fraction of lipids functionalized with the Ni-NTA group. Elementary building blocks encapsulated in the vesicle consist of actin and polyhistidine-tagged anillin cross-linker, which is sufficient for the formation of cytoskeletal network coupled to the lipid membrane via the Ni-NTA lipid/his-anillin links. (*b*) A 3D reconstruction of the cytoskeletal vesicles produced using cDICE shows the close proximity of the actin cortex to the membrane. A section of the membrane has been masked to show the underlying actin cortex. The scale bar represent 5 *μ*m. (*c*) When myosin motors are added to the vesicles, they induce small dynamic shape deformations, as indicated by the white arrows. A membrane labeled with Texas Red has been used in combination with epifluorescence to record these deformations. The scale bars represent 5 *μ*m. (*d*) To compare the membrane fluctuations, intensity line profiles across the section of the membrane were used in the Fiji plugin to prepare the kymographs. The three kymographs show that a cortex-free vesicle has more prominent fluctuations in the membrane than a passive or active vesicle. The vesicles chosen for preparing kymographs were of size 17, 19, and 20 *μ*m for cortex free, passive, and active, respectively. (*e*) The schematic shows the scheme that we have adopted to attach vesicles to the glass surface. The PEG2000PE lipids in the membrane prevent the nonspecific electrostatic interaction between the membrane and the glass surface. To see this figure in color, go online.

Our model system is a giant unilamellar vesicle containing a cross-linked actin cortex anchored to its inner leaflet. The His-tagged anillin is responsible for both cross-linking the actin and coupling the actin network to the Ni-NTA lipids that are incorporated into the membrane. We call the vesicle that has an actin cortex a cytoskeletal vesicle, as shown in Fig. 1, *a* and *b*. Protein encapsulation occurs during vesicle preparation using the cDICE method adapted for this system (18). By adding myosin motors to the network, we added contractility and hence activity. Depending on the presence or absence of motor proteins, we call cytoskeletal vesicles active vesicles or passive vesicles, respectively. The actin cortex is formed by encapsulating 10 *μ*M of G-actin and 1.5 *μ*M of anillin at 4°C. We induced contractility into the cortex by adding an additional 0.1 *μ*M of myosin motors into the reaction mix. The amount of anillin inside the vesicle and mol % of Ni-NTA needed to form an actin cortex has been characterized in depth in previously published work (18). The active vesicles were observed having dynamic deformations (Fig. 1 *c*; Videos S1 and S2), unlike the passive and cortex-free vesicles. These deformations are due to the active stress generated by the myosin motors in actin cortex. Myosin motors are known to create sliding motion between the actin filaments. It is this sliding of the filaments and tension in the membrane that causes dynamic shape changes in case of the active vesicles (Fig. S2). The characteristic timescale over which we observed the active shape changes is much larger than the timescale of membrane fluctuations. The active cortex pushes and pulls on the lipid bilayer, causing tiny vertices to appear on the vesicle surface approximately once every 20 s (Video S2). In contrast, the membrane fluctuations in the cortex-free vesicles are of a much higher frequency, 2 s^−1^, as is evident from the kymograph in Fig. 1 *d*. No microscopic membrane fluctuations, as observed in the cortex-free vesicles, were seen in the cytoskeletal vesicles. This can be best seen by comparing the kymographs shown in Fig. 1 *d*. The kymographs were obtained from the line intensity profiles taken across a section of the membrane from the epifluorescence time-lapse recording of the vesicles with labeled membrane. The absence of the microscopic fluctuations can be attributed to anchoring of the elastic actin cortex to the membrane, which kills the high-amplitude fluctuation modes. These observations already indicate that the excess membrane area is strongly coupled to the actin cortex and is no longer free for shape transformations in cytoskeletal vesicles.

Video S1. Active Cytoskeletal Vesicle The active cytoskeletal vesicle when not attached to the surface and is in the suspension exhibits shapes deviated from a perfect sphere. This can be attributed to the motor activity included in the actin cytoskeleton as the passive vesicles did not show such behaviour. The z-stacks shown in the two rows clearly show that the actin cortex lies just beneath the membrane and is behind the observed deformations. The scale bars are 5 μm.

Video S2. Active Shape Remodeling Though the active cytoskeletal vesicle lack membrane undulations, a continuous remodeling of the cortex can be seen in the form of small but dynamics shape deformations. The membrane is labeled with Texas red to visualize the deformations caused by active remodeling of the cortex. Vortices can be seen appearing and disappearing at the surface of the vesicle due to the cortex activity. The time shown in the time lapse movie is in seconds.

### Adhesion of cytoskeletal vesicles

The specific adhesion strength between the vesicle and the functionalized glass surface can be controlled by tuning the ligand-receptor density between the two. We used biotin-streptavidin as a ligand-receptor pair to make vesicles adhere to the glass (Fig. 1 *e*). We used two different ligand densities at the glass surface by coating the glass with BSA-biotin and BSA mixed at two different volume ratios, 70:30 and 50:50. The membrane was doped with 1 mol % PEG-biotin lipids to make the vesicles bind to streptavidin on the glass surface.

**Figure 2:**
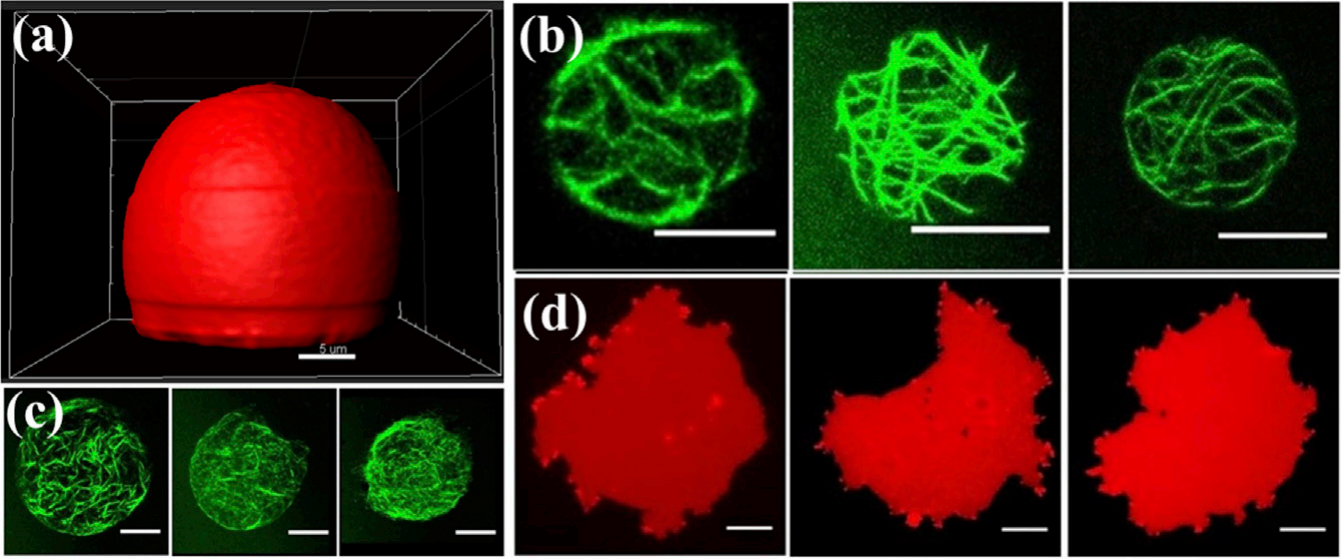
(*a*) The spherical cap shape adopted by the cortex-free vesicle under strong adhesion. The membrane was labeled with Texas Red to visualize the membrane for confocal scanning. The scale bar represents 5 *μ*m. (*b*) The bottom-most slice from the z-stacks of three different active vesicles that did not burst even after 30 min of contact with the functionalized glass surface. (*c*) A collapsed actin structure of three different active vesicles that burst on the surface after making contact. Scale bars, 10 *μ*m. (*d*) Three different active vesicles that bursted, showing the formation of supported bilayer with an irregular shape on the surface. The membrane was labeled with Texas Red. The scale bar represents 10 *μ*m. To see this figure in color, go online.

We observed that for both ligand densities the cortex-free vesicles bind to the rigid glass surface and adopt a spherical cap shape (Fig. 2 *a*). 5% of vesicles get leaky but maintain their shape, as can be seen in Fig. S3. We excluded all the leaky vesicles from further analysis. As per the previously published theoretical estimates, a vesicle with a constant volume adopts a spherical cap shape under strong adhesion conditions (10). It has also been shown that the contact angle provides a measure for the adhesion strength (28, 29, 30). We adopted the same approach for concluding if a vesicle is strongly adhered or not. Therefore, if a vesicle makes an acute angle (<90°) with the surface, we call it a strong adhesion; otherwise, we consider the adhesion weak. We used Fiji to determine the contact angles from the 3D projections of the confocal stacks as can be seen in Fig. S4. On a glass surface coated with a mix of 70% BSA-biotin and 30% BSA, all cytoskeletal vesicles burst within a minute after making contact with the surface. The passive vesicles have their membrane area bound to the cortex which makes it difficult for the lateral forces to pull the excess membrane area to gain in adhesion energy. In the case of the active vesicles, the contractile forces in the cortex increase membrane tension, leading to a membrane rupture under adhesion.

On lowering the ligand density on the surface (50% BSA-biotin and 50% BSA), we observed that some size selection occurs and cytoskeletal vesicles with a longest chord length shorter than 20 *μ*m are stable for more than 30 min. We used the longest chord length as a way to determine the vesicle size because the vesicles deviate from a spherical shape when they make contact with the surface. Fig. 2, *b* and *c* show the bottom slice from the z-stack of active vesicles that did not burst against the vesicles that burst after making contact with the surface, respectively. Vesicles that burst after contact with the surface form an irregular supported bilayer visible only in the bottom-most slice of the z-stack (Fig. 2 *d*). Our observations show that the vesicles that do not burst after 30 min of contact with the glass surface form contact area with a diameter ≤14.4 ± 1.9 *μ*m (mean ± SD; *n* = 20). Bigger vesicles form a larger contact area before they burst, which can be seen in the footprint of the active vesicles that burst in Fig. 2 *c*. One possible reason for the observed size dependency is the local curvature of the vesicles at the contact area, which determines accessibility of the binding partners. The accessibility of the binding partners increases with decreasing curvature (increasing radius) and hence the attractive forces between the vesicle and the glass surface. The increased attraction will consequently lead to increased lateral forces. Because the limiting parameter here would be the deformability of the vesicle and the membrane is bound to the cortex underneath, we conclude that the elasticity of the cortex limits the spreading dynamics. In the case of vesicles with diameters >20 *μ*m, the large membrane curvature generates lateral forces, which increase the membrane tension beyond the lysis limit, causing the cytoskeletal vesicles to burst.

The finding that the cytoskeletal vesicles burst, whereas cortex-free vesicles are able to adhere at a similar ligand-receptor density, can be attributed to the difference in the availability of excess membrane. Cortical coupling has already been reported to limit available excess area and thus limits the vesicle’s ability to form membrane tubes under hydrodynamic flow (31). To increase adhesion, a vesicle needs to deform, which is only possible at the cost of excess membrane area and increase in the membrane tension. Membrane tension limits gain in adhesion once the excess membrane area has been consumed. Owing to the coupling of membrane to the cortex, the excess membrane area is not freely available to make deformations in cytoskeletal vesicles. Therefore, the increase in contact area beyond the cutoff (∼15 *μ*m) causes the lysis of the membrane in these vesicles. In the next series of experiments, we aimed to increase the available excess area by applying hypertonic osmotic stress to enable the strong adhesion of cytoskeletal vesicles.

### Deformation of cytoskeletal vesicles under hypertonic osmotic stress

**Figure 3:**
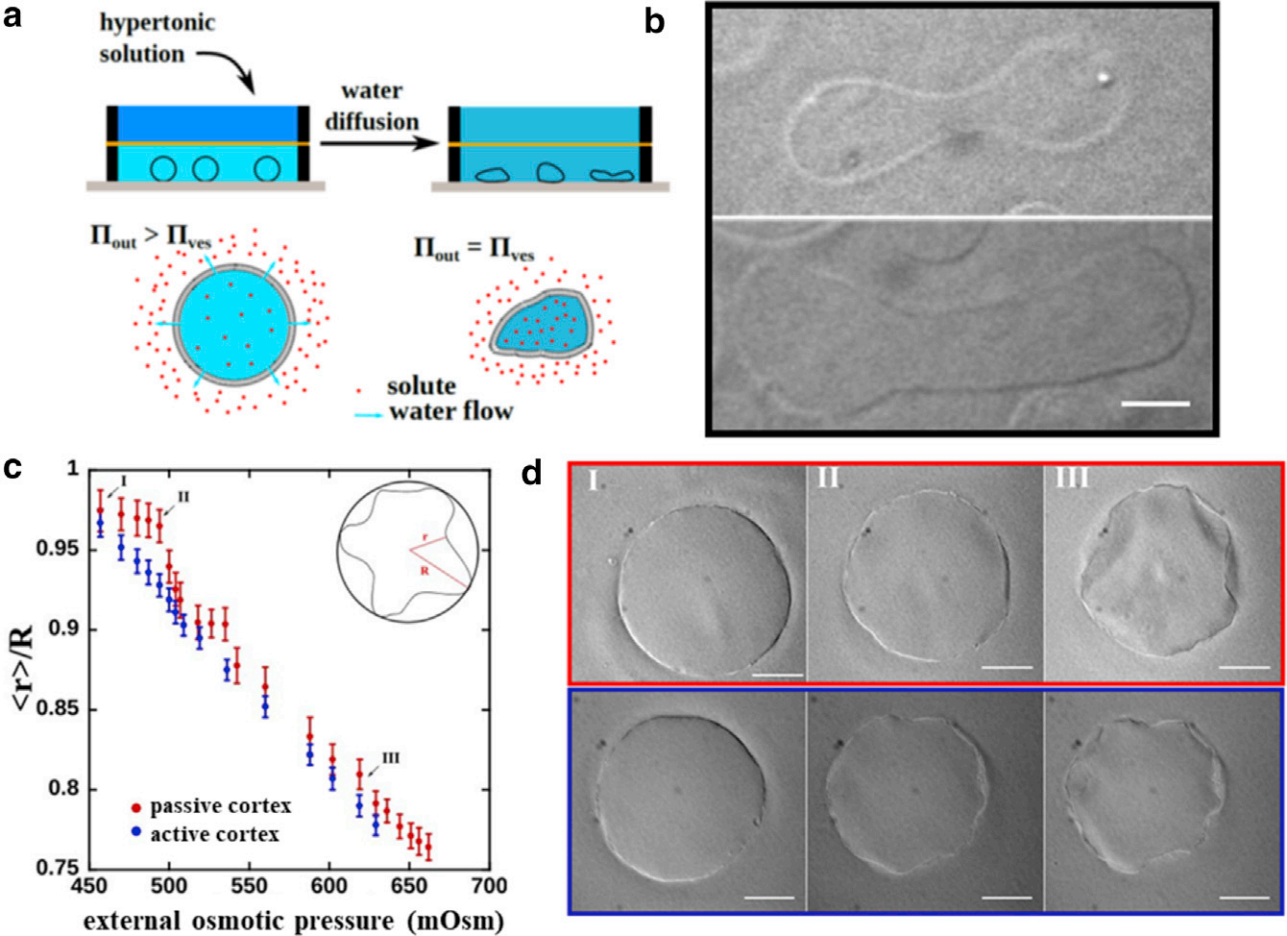
(*a*) The two-leveled chamber used to deflate the vesicles. The top chamber was filled with glucose solution with a higher osmolarity than the solution encapsulated inside the vesicle. To image the vesicles, the entire chamber was closed and put on the microscope stage. (*b*) Deflation of the cortex-free vesicles shows the well-documented shape transformations due to the Helfrich-energy minimization. (*c*) For the passive cytoskeletal vesicles, the change in external osmotic pressure does not immediately cause any shape remodeling (from point I to II) but causes a continuous shape remodeling for active vesicles (from point I to III). (*d*) Phase-contrast images of passive (*red panel*) and active (*blue panel*) cytoskeletal vesicles at time points indicated in (*c*). The active vesicle is able to remodel the cytoskeleton and deforms actively while the pressure changes from I to II, whereas the passive vesicles resist the increasing osmotic pressure at first. At point III, both vesicles, the passive and the active ones, have reached a highly deformed shape with similar reduced volumes. Similar deformed shapes are observed for both the active and the passive vesicles although via different trajectories. For the passive vesicles, deformations appear abrupt, whereas for active vesicles, a continuous deformation is observed. The curves in (*c*) are the average values taken over five vesicles for the passive case and eight vesicles for the active case. The error bars are the SDs on the measure of the mean contracted radius <*r*>. We computed the Kruskal-Wallis test at the point II and second step (right before the passive vesicles crumple). The *p*-value comparing the data sets of active and passive vesicles are *p* = 0.032 and *p* = 0.044, respectively. Scale bars, 20 *μ*m. To see this figure in color, go online.

Because the limiting parameter for creating adhesion is a lack of excess membrane area, we applied a hypertonic osmotic stress using a two-level diffusion chamber (Fig. 3 *a*) to deflate the vesicles to a reduced volume of *ν* = 0.6 (40% volume loss). The reduced volume is defined by the ratio between the volume of liquid present in the deformed vesicle and the volume enclosed by a sphere with the same surface area. We compare the deformations of passive and active vesicles in the nonadhering state under hyperosmotic pressure to pinpoint the effect of myosin motors on shape adaptations. Cortex-free vesicles show the well-described morphological deformations predicted by the minimization of curvature energy of the lipid membrane (32, 33, 34) (Fig. 3 *b*; Video S3). In contrast, the passive cytoskeletal vesicles remain mostly spherical for up to a 10% increase in the osmotic pressure without changing their volume (Fig. 3 *c*). Because the volume of the passive vesicle does not change significantly, we estimate that the resulting applied pressure reaches 0.11 atm. The reduced volume and the vesicle shape remain almost the same as the surrounding osmotic pressure increases from 450 to 500 mOsm (Fig. 3, *c* and *d*). Increasing the osmotic pressure further leads to a further compressive stress buildup and finally to an abrupt deformed shape change (∼6% radius decrease). After this abrupt deformation, when the pressure reaches 520 mOsm, the cortex stability again resists further deformations until the pressure exceeds 540 mOsm (Fig. 3 *c*) and a second sudden shape change occurs. After the second sudden event of cortex shape change, the radius of the vesicle starts decreasing monotonically as external pressure increases.

Video S3. Deflated Cortex-Free Vesicles The cortex free vesicles shown well studied deformations when subjected to hyper osmotic stress.

In contrast to these discontinuous deformations of the passive cytoskeletal vesicles, the presence of 0.1 *μ*M of myosin motors enable vesicles to adapt continuously to the osmotic pressure change. Indeed, the myosin contractile activity pulls on the membrane, and the limiting parameter to deformation is now the membrane tension of the vesicle. We observe a continuous remodeling of vesicle shape, without any sudden instabilities (Fig. 3 *b*). The final equilibrium shape of both passive and active vesicles is comparable (Fig. 3 *c*). Both vesicle types show a complex morphology with many invaginations, resembling a crumpling transition of an elastic shell (Fig. 3 *c* at point III; Fig. S5; Video S4), as predicted for spherical elastic shells submitted to a constant compressive rate (35).

Video S4. Deforming Cytoskeletal Vesicles Under the condition of hyper osmotic stress, both active and passive vesicles undergo shape deformations. These deformations are discontinuous in case of the passive vesicles and continuous for the active vesicles. This is due to the active remodeling of the cortex in active vesicles.

### Adhesion of cytoskeletal vesicles under hypertonic osmotic stress

**Figure 4:**
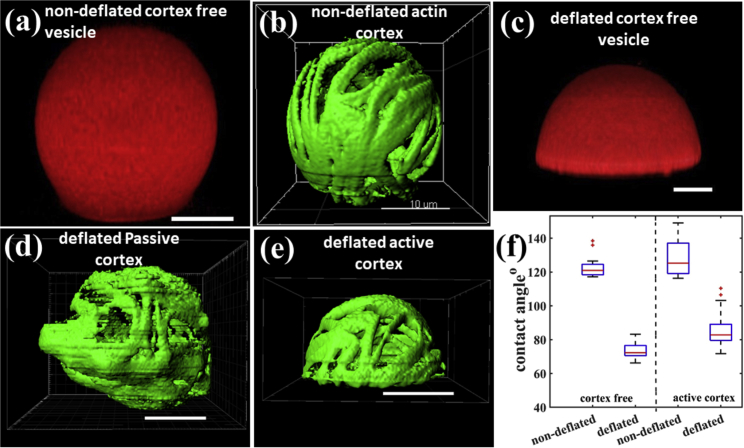
(*a* and *b*) Cortex-free and passive cytoskeletal vesicle in the weakly adhered or nondeflated state, respectively. (*c*) The cortex-free vesicle adopts a spherical cap shape after deflation, a signature of strong adhesion. (*d* and *e*) The passive and active vesicles, respectively after deflation. The deformation leads to undefined shapes in the case of the passive vesicles and spherical cap shape in active vesicles. (*f*) The almost equal difference in the contact angle between nondeflated and deflated state for cortex-free and the active cortex vesicles shows that the active vesicle can use the excess membrane area developed by deflation in reducing the contact angle to spread on the surface. 20 vesicles were imaged for each vesicle type for the box-and-whisker diagram. The method we used to estimate the contact angle is shown in the Fig. S4. Scale bars, 10 *μ*m. To see this figure in color, go online.

Because adhesion depends on the availability of excess area, we tested the adhesion process of active and passive cytoskeletal vesicles having diameters larger than 20 *μ*m under hypertonic stress. To avoid immediate bursting of vesicles, we slightly reduced the adhesion strength between the membrane and the glass by adding 0.5 mol % PEG2000 lipids to the membrane and by coating the glass with a mix of 35% biotin-BSA and 65% BSA instead of a 50:50 mix. Cytoskeletal vesicles are still unstable and observed to burst even under the lower adhesion strength. Because of the lower density of adhesion molecules on the surface and the presence of PEG lipids in the bilayer, vesicle adhesion slows down and the membrane rupture is delayed by around 10–15 min. Because assembling of the diffusion chamber takes ∼1–2 min, the cytoskeletal vesicles are under hypertonic stress and start to deflate long before they can reach the previously observed lysis point. To estimate the contact angle in a nondeflated state, we performed control experiments. In the control experiments, we acquired z-stacks of 20 vesicles in the first 10 min of the adhesion process under no osmotic stress. Fig. 4, *a* and *b* represent the typical shape of a cortex-free and a cytoskeletal vesicle in the nondeflated state, respectively. A z-stack of a cytoskeletal vesicle before deflation can be seen in Video S5. We found no difference between the shapes of passive and active vesicles before deflation, and the average contact angle was found to be around 122° for all the different types of vesicles. We chose the glucose concentration in the top chamber to be 570 mOsm higher than the bottom chamber to get larger deformations in a shorter time than the nonattached vesicles. The reduced vesicle volume in this case was around 48%. In 60 min, the solution in the two compartments reached equilibrium (calibration plot in Fig. S1) and we started imaging.

Video S5. Weakly Adhered Cytoskeletal Vesicle The video shows z-stack of a weakly adhered cytoskeletal vesicle with actin in green channel and membrane in red. The scale bar here is 5μm. The bright green illumination is the reflection from the surface. The image represents raw data without any image processing and hence serves as a proof of actin cortex lying beneath the lipid bilayer. There was no difference found between the geometry/shape of an active and a passive vesicle in the weakly adhered state.

We observed that the deflated cytoskeletal vesicles (both active and passive) remain stably adhered to glass even after 2 h of coming in contact with the glass without any observable bursting events. We observed cortex-activity-dependent shape transformations in cytoskeletal vesicles under hypertonic stress. After a volume reduction of around 48%, passive vesicles became strongly and irregularly deformed (Fig. 4 *d*; Video S6), whereas all adhered active vesicles adopted the shape of a smooth spherical cap (Fig. 4 *e*; Video S7). We observed that after deflation, the contact angle and shape of the active vesicles are identical to those of cortex-free vesicles (Fig. 4, *c*, *e*, and *f*; Video S8). The identical shape transformation and change in contact area exhibited by the cortex-free vesicles and the active cytoskeletal vesicles after deflation suggest that excess membrane area is created by active deformation of the vesicles to increase adhesion to the surface and to reduce the contact angle. For both cortex-free vesicles and active cytoskeletal vesicles, the contact angle changes from ∼122° in the nondeflated state to ∼70° in the deflated state, as shown in Fig. 4 *f*. Thus, active remodeling of the cortex is needed to get the desired shape transformations that can lead to a gain in the adhesion area. Passive vesicles lack the ability to actively remodel their cortex and hence just crumple under the osmotic pressure change.

Video S6. Passive Vesicle on Adhesive Surface under Hyperosmotic Stress The video shows z-stack of a passive vesicle taken at time when the inside and outside buffer reaches an equilibrium. Actin is in green channel and membrane in red. The scale bar here is 5μm. The bright green illumination in the beginning is from the reflection from the glass surface. The contrast of the images has been enhanced using ImageJ plugin to have a better visualization of the top slices of the z-stack. Other than an undefined shape the passive vesicles exhibit either the formation of tubes (a) or invaginations (b) in the membrane. The invaginations are observed if the vesicle after deswelling shows a gain in contact area.

Video S7. Active Vesicle on Adhesive Surface under Hyperosmotic Stress The video shows z-stack of an active vesicle after the inside and outside buffer reaches an equilibrium. Actin is in green channel and membrane in red. The scale bar here is 5μm. The contrast of the images has been enhanced using ImageJ plugin to have a better visualization of the top slices of the z-stack.

Video S8. Adhesion under Hyperosmotic Stress The video shows 3D projections of the actin cortex inside cytoskeletal vesicles. The weekly adhered cytoskeletal vesicles (left most) undergo shape deformation when in hyper osmotic stress. In case of the passive vesicles ( in the middle) these deformations do not get pulled out by the lateral forces from the surface but in case of the active vesicles (right most), the deformations can be pulled out to gain in adhesion strength and a well-defined spherical cap geometry. This shows that it is only in the presence of active remodelling of the cortex that the membrane area in the deformations can be pulled out by the lateral forces to let the vesicle spread on the surface.

The resulting shapes of the passive vesicles in nonadhered and adhered conditions are indistinguishable. It demonstrates that under experimental conditions presented here, adhesive forces alone are not sufficient to induce a shape change in the passive elastic shell. The contact angle of passive vesicles could not be determined after deflation because of its highly deformed random shape near the surface. A comparison between the shape acquired by a weakly adhered passive and an active vesicle after 48% volume reduction can be seen in Video S8.

As discussed in the previous section, passive vesicles show abrupt changes in their shape caused by the sudden crumpling of the elastic actin cortex. For nonadhering passive vesicles, we observed that the cortex crumples only when the osmotically induced deformation forces are sufficiently high (Fig. 3 *b*). In comparison, the attractive forces from the small adhesion zone are too small to induce any shape remodeling in passive vesicles. Consequently, any excess area resulting from deflation remains trapped as randomly distributed invaginations and is not available to increase the adhesion area.

In the case of active cytoskeletal vesicles, the presence of myosin motors develops activity in the cortex and enables cortical remodeling. The osmotic pressure and the adhesion forces both are able to induce deformations. During adhesion-area formation, the osmotic pressure continuously yields sufficient excess area, which is then continuously pulled laterally by the adhesive molecules. Excess area of the osmotically induced deformations is thus made available for adhesion by the motor activity. Our experiments show that the availability of excess membrane area depends on the ability of the actin cortex to remodel actively.

## Conclusion

Our observations highlight the mechanism of balancing between cortex attachments, cortex remodeling, and adhesion-induced contact-area formation. We present a model system that we have used to explore the complex interplay between membrane tension, cytoskeleton elasticity, and active forces in the context of specifc adhesion. Upon adhesion to a rigid substrate, cytoskeletal vesicles need to accommodate the shape of the coupled cytoskeleton/membrane shear elastic material. Whereas the lipid membrane is fluid and nonstretchable, the elastic cytoskeleton can be sheared and stretched. These two mechanical properties result in a strong constraint for the system. Cystoskeletal vesicles can withstand strong adhesion provided that two conditions are fulfilled. First, some excess membrane area must be available to allow shape remodeling without overcoming the critical lysis membrane tension; this is evidenced by the fact that large cytoskeletal vesicles with the surface/volume ratio of a sphere burst when starting to adhere. Second, the actin cortex should be able to undergo remodeling to accommodate the substrate configuration. Under the experimental conditions of our study, molecular motors generate active forces to dynamically remodel the cytoskeleton. In the case of a passive cortex, its stiffness prevents the vesicle from spreading and adhering to a substrate even in the presence of excess membrane area provided by increasing the surface/volume ratio. The work presented here provides a conceptual framework for further investigation of the interplay between the formation of adhesion patches and cytoskeletal remodeling in a more realistic environment in which adhesion molecules have finite lifetimes and the actomyosin cortex shows a turnover behavior. These will be essential steps toward building a more complex biomimetic system to help us understand the underlying principles of the physics governing the formation and dynamics of cellular adhesion.

## Author Contributions

R.M., E.L., and A.R.B. planned the experiments. R.M. and E.L. performed the experiments and analyzed the data. R.M., E.L., and A.R.B. wrote the article.

## Supporting Material

Document S1. Supporting Materials and Methods and Figs. S1–S5

Document S2. Article plus Supporting Material
