# Supplementary material for: Adhesion of Active Cytoskeletal Vesicles
Source: Biophys J. 2018 Oct 23;115(12):2395–402. doi: 10.1016/j.bpj.2018.10.013 (PMC6301914; doi:10.1016/j.bpj.2018.10.013)
Supplement: Document S1. Supporting Materials and Methods and Figs. S1–S5 [file mmc1.pdf]

Biophysical Journal, Volume *115*

Supplemental Information

Adhesion of Active Cytoskeletal Vesicles

Renu Maan, Etienne Loiseau, and Andreas R. Bausch

Figure S1: Osmotic chamber calibration

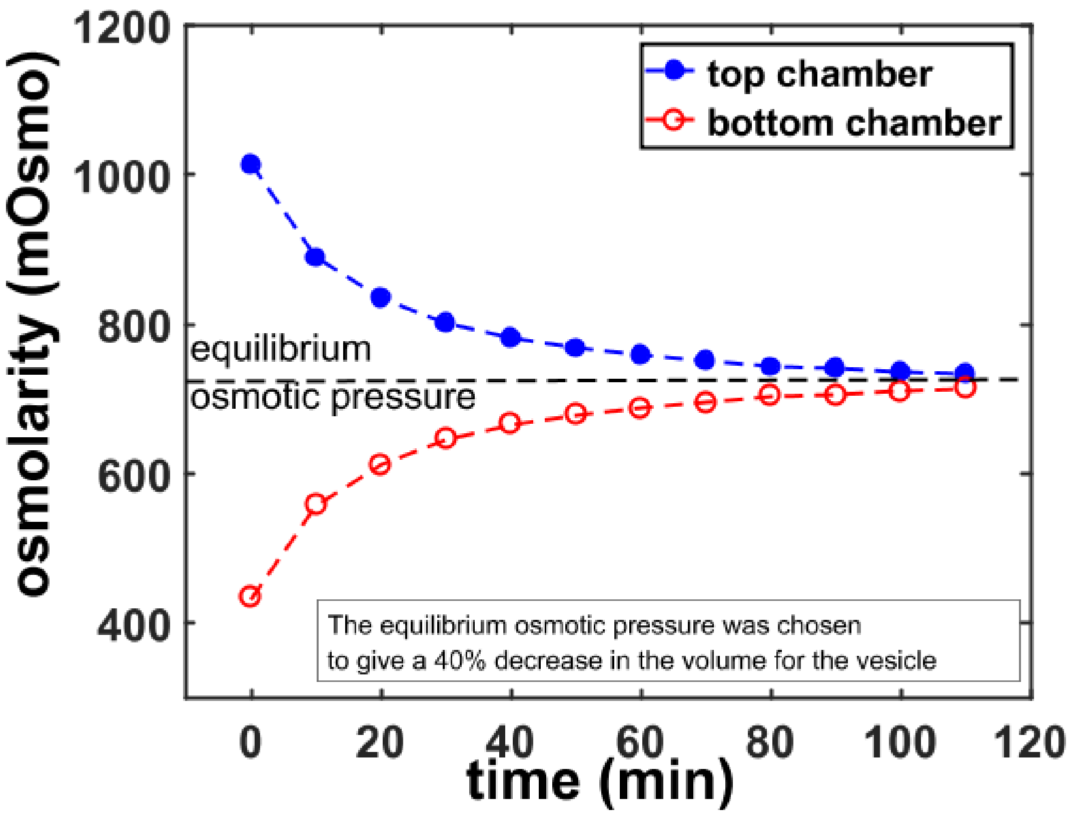

Figure S1: Osmotic chamber calibration. The top chamber of the two level osmotic chamber was filled with 1M Glucose solution. As the two chambers are separated by the membrane the glucose from the top chamber diffuse into the bottom one and the osmotic pressure of the top chamber decreases. The osmotic pressure of the top chamber is measured after every 10 minutes by collecting 50 µl of the solution from the top chamber and plotted against the time. The solution collected was added back to the chamber to avoid differences in volume of the solution in the top chamber. The plot shows that the diffusion is very fast in the first 10—20 minutes and equilibrate after around 60—70 minutes.

Figure S2: Active deformations

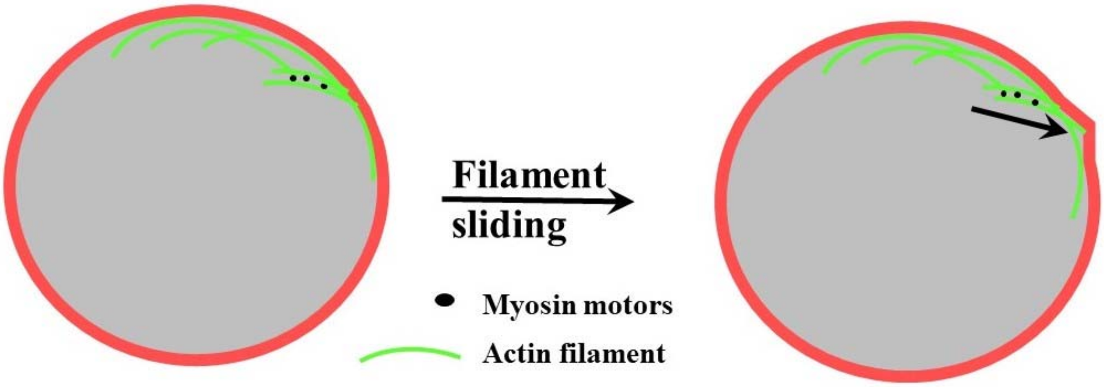

Figure S2: Active deformations. In the 2d cortex the myosin motors exert contractile forces. When myosin slides two actin bundles towards each other this can results in a situation where the end of the bundle pokes in the membrane as shown in the schematic. Sliding of the filaments can also cause pulling of the membrane in the similar way. The pulling and pushing of the membrane is therefore a result of actin reorganization which is supported by myosin activity.

Figure S3: Leaky vesicles

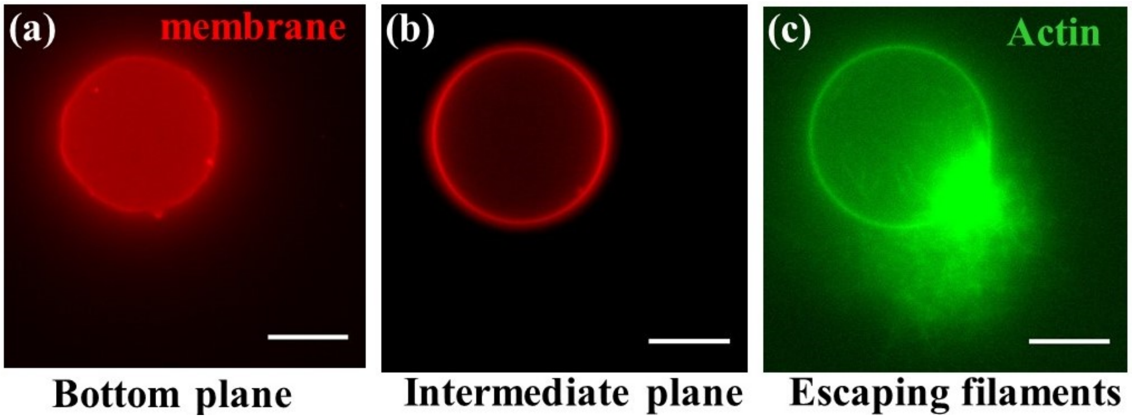

Figure S3: Leaky Vesicles. The membrane integrity of the cortex free vesicles was tested by adhering the cortex free vesicles to the surface with high ligand-receptor density. We observed that around 2—3 vesicles in 30—40 will show leaky membrane. (a) The bottom most plane taken from the z-stack of a leaky vesicle. (b) The intermediate plane from the z-stack of the leaky vesicle. (c) 10 µM actin with the polimerization buffer was encapsulated in the vesicles without the cross linker. Since in our experiments, it is the actin crosslinker Anillin that binds the actin to the membrane, the cortex will not be formed. The actin filaments remain in the solution and in case of a rupture in the membrane can be seen flowing out. Actin was labelled using 0.5 µM phalloidin and membrane with 0.1 mol% PE lipids with Texas red. The scale bar in the figure is 5 µm.

Figure S4: Contact angle estimation

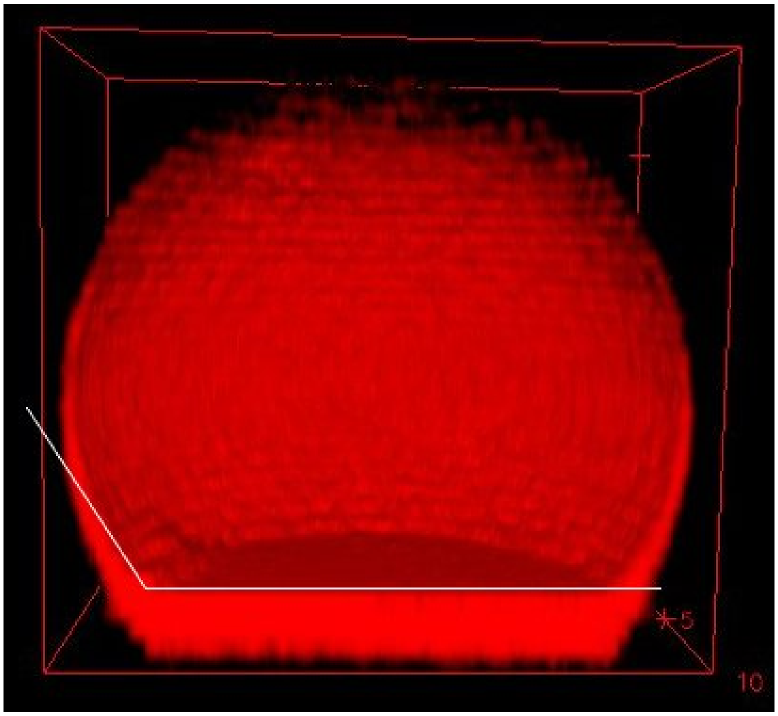

Figure S4: Contact angle estimation. 3D reconstructs of the vesicles were prepared using Fiji Plugin in such a way that only half of the vesicle is reconstructed. From such hemisphere, using imageJ, we estimated the contact angles of the cortex free vesicles and the active vesicles.

Figure S5: Drastic shape deformations

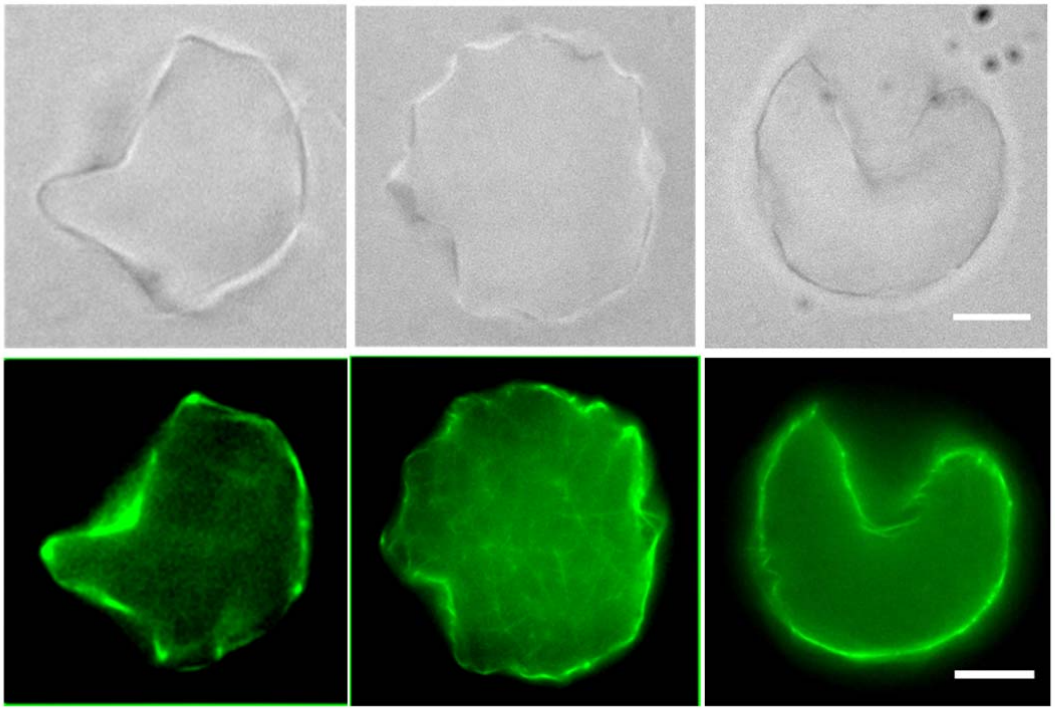

Figure S5: Drastic Shape deformations. Vesicles with cortex can go to drastic shape deformations under the osmotic pressure depending on the non-homogeneity of the actin cortex beneath the membrane. The figure shows three individual examples of such deformations in the cytoskeletal vesicles. Top panel shows the bright field images of the vesicles with corresponding labelled actin cortex in the bottom panel. The actin here is labelled using Phalloidin 488 and the scale bar in the figure is 20µm.

Supporting Movie S1: Active cytoskeletal Vesicle. The active cytoskeletal vesicle when not attached to the surface and is in the suspension exhibits shapes deviated from a perfect sphere. This can be attributed to the motor activity included in the actin cytoskeleton as the passive vesicles did not show such behaviour. The z-stacks shown in the two rows clearly show that the actin cortex lies just beneath the membrane and is behind the observed deformations. The scale bars are 5 µm.

Supporting Movie S2: Active shape remodeling. Though the active cytoskeletal vesicle lack membrane undulations, a continuous remodeling of the cortex can be seen in the form of small but dynamics shape deformations. The membrane is labelled with Texas red to visualize the deformations caused by active remodeling of the cortex. Vortices can be seen appearing and disappearing at the surface of the vesicle due to the cortex activity. The time shown in the time lapse movie is in seconds.

Supporting Movie S3: Deflated cortex free vesicles. The cortex free vesicles shown well studied deformations when subjected to hyper osmotic stress.

Supporting Movie S4: Deforming cytoskeletal vesicles. Under the condition of hyper osmotic stress, both active and passive vesicles undergo shape deformations. These deformations are discontinuous in case of the passive vesicles and continuous for the active vesicles. This is due to the active remodeling of the cortex in active vesicles.

Supporting Movie S5: Weakly adhered cytoskeletal vesicle. The video shows z-stack of a weakly adhered cytoskeletal vesicle with actin in green channel and membrane in red. The scale bar here is 5µm. The bright green illumination is the reflection from the surface. The image represents raw data without any image processing and hence serves as a proof of actin cortex lying beneath the lipid bilayer. There was no difference found between the geometry/shape of an active and a passive vesicle in the weakly adhered state.

Supporting Movie S6: Passive vesicle on adhesive surface under hyper osmotic stress. The video shows z-stack of a passive vesicle taken at time when the inside and outside buffer reaches an equilibrium. Actin is in green channel and membrane in red. The scale bar here is 5µm. The bright green illumination in the beginning is from the reflection from the glass surface. The contrast of the images has been enhanced using ImageJ plugin to have a better visualization of the top slices of the z-stack. Other than an undefined shape the passive vesicles exhibit either the formation of tubes (a) or invaginations (b) in the membrane. The invaginations are observed if the vesicle after deswelling shows a gain in contact area.

Supporting Movie S7: Active vesicle on adhesive surface under hyper osmotic stress. The video shows z-stack of an active vesicle after the inside and outside buffer reaches an equilibrium. Actin is in green channel and membrane in red. The scale bar here is 5µm. The contrast of the images has been enhanced using ImageJ plugin to have a better visualization of the top slices of the z-stack.

Supporting Movie S8: Adhesion under hyper osmotic stress. The video shows 3D projections of the actin cortex inside cytoskeletal vesicles. The weekly adhered cytoskeletal vesicles (left most) undergo shape deformation when in hyper osmotic stress. In case of the passive vesicles (in the middle) these deformations do not get pulled out by the lateral forces from the surface

but in case of the active vesicles (right most), the deformations can be pulled out to gain in adhesion strength and a well-defined spherical cap geometry. This shows that it is only in the presence of active remodelling of the cortex that the membrane area in the deformations can be pulled out by the lateral forces to let the vesicle spread on the surface.
